# Case Report: Nephrocalcinosis in an infant due to vitamin-D food supplement overdose

**DOI:** 10.3389/fped.2024.1485814

**Published:** 2024-11-19

**Authors:** Carla Pizzini, Andrea Ossato, Nicola Realdon, Roberto Tessari

**Affiliations:** ^1^ Department of Pediatrics, IRCCS Ospedale Sacro Cuore Don Calabria, Negrar di Valpolicella, Italy; ^2^ Department of Pharmaceutical and Pharmacological Sciences, University of Padova, Padova, Italy; ^3^ Hospital Pharmacy, IRCCS Ospedale Sacro Cuore Don Calabria, Negrar di Valpolicella, Italy

**Keywords:** nephrocalcinosis, vitamin D, calciferol, food supplement, hypercalcaemia

## Abstract

**Background:**

Vitamin D is a vital lipophilic vitamin that plays a pivotal role in calcium regulation, bone metabolism, and overall health. It is of the utmost importance to maintain appropriate serum levels of vitamin D from the moment of birth. The recommended daily intake for infants under the age of 12 months is 400 IU. In Europe, vitamin D is available in two forms: as a medicinal product and as a food supplement. The food supplement market is experiencing rapid growth, yet it is characterised by a lack of harmonised regulations, which may give rise to potential risks associated with their widespread use. While food supplements are typically regarded as safe, there is a potential for adverse effects, particularly when dosages are not properly managed.

**Case report and management:**

This report presents the case of a 22-month-old girl who developed nephrocalcinosis as a result of an overdose of vitamin D from a dietary supplement purchased online. The initial presentation was characterised by symptoms such as polydipsia, polyuria and decreased growth. It was subsequently revealed that the child had been receiving an excessively high dose of vitamin D, amounting to 25 times the recommended amount, over a period of seven months. Despite normal calcium levels and renal function at the time of presentation, ultrasound imaging revealed the presence of early-stage nephrocalcinosis. The treatment plan involved hospital admission, intravenous hydration, a thiazide diuretic, potassium citrate, and a low-calcium diet. The vitamin D supplement was ceased. Over the course of a year, the patient demonstrated recovery in growth and normalization of vitamin D levels, although nephrocalcinosis remained stable.

**Conclusion:**

This case study highlights the potential dangers of unsupervised vitamin D supplementation, emphasising the importance of healthcare professionals exercising vigilance in prescribing and advising on vitamin D use, particularly in children. Furthermore, it underscores the necessity of establishing a database to track long-term outcomes in paediatric vitamin D intoxication cases, given the rarity of such incidents. This would facilitate the development of appropriate treatment protocols and provide valuable information to parents.

## Introduction

Vitamin D (calciferol) is a very important lipophilic vitamin involved in calcium homeostasis, bone metabolism, immune system regulation, cardiovascular and musculoskeletal health and other secondary functions (1, 2). Its serum level is closely monitored from birth, to keep it within the optimal range for age and various physio-pathological conditions (serum 25-hydroxyvitamin D≥20 ng/ml). For this reason, the international consensus on recommended dietary intake of vitamin D for children under 12 months of age is 400 IU/day (3–5).

Vitamins, and in particular vitamin D, are available in Italy either as a “medicinal product” or as a “food supplement”. In Europe, the food supplements market is growing rapidly and is under the General Food Regulation [Regulation (EC) No 178/2002] (6).

They consist of nutrients like minerals and vitamins, as well as other substances such as herbal extracts. They are distinct from medicinal products, as their purpose is not to treat or prevent diseases pharmacologically, but rather to aid specific physiological functions, claiming a physiological effect. Moreover, despite the absence of a specific European regulation harmonization, these supplements are widely accessible throughout Europe due to free cross-border movement and mostly to the strong online market (7).

To date, although food supplements are widely used by consumers, the only tools available to increase awareness of the risks associated with their use are voluntary adverse reaction reporting systems; however, only some European countries have established dedicated surveillance systems for such reactions (i.e.,: vigierbe in Italy; https://www.vigierbe.it/). However, these systems capture a limited number of adverse reactions related to food supplements. This limitation is exacerbated by the perception among consumers and health professionals that these products are “natural” and safe, rather than something that might be poisonous, leading to an underestimation of potential risks as recently reported in literature (1, 2, 8–12).

Vitamin D toxicities appear to be unfrequently but can have a wide spectrum of clinical manifestation, according to recent reviews and case reports. In particular, excessive doses of vitamin D, is associated with hypercalcemia, hypercalciuria, kidney stone, altered bone metabolism, weight loss, polyuria, diarrhea, dehydration, etc.… and, in extreme and rare cases, deposition of calcium phosphate crystals in soft tissues throughout the body, cardiac abnormalities, nephrocalcinosis and renal failure (9). Common causes include the use of unlicensed and poorly manufactured products, the widespread availability and inappropriate use: overdose by patients or prescribers, and combinations of these factors (2, 8).

Nephrocalcinosis, previously known as Anderson-Carr disease or Albright calcinosis, is a pathological condition characterized by the deposition of calcium salts within the kidney's parenchyma. It is classified into various stages, distinguished by their underlying causes and distribution patterns. Medullary nephrocalcinosis represents 95% of cases, whereas cortical nephrocalcinosis accounts for 5% (13). The following case study presents an infant with nephrocalcinosis secondary to an overdose of a vitamin D supplement purchased online. A detailed description of this particular case will be provided, along with an in-depth analysis of the long-term follow-up period.

## Case description

**Figure 1:**
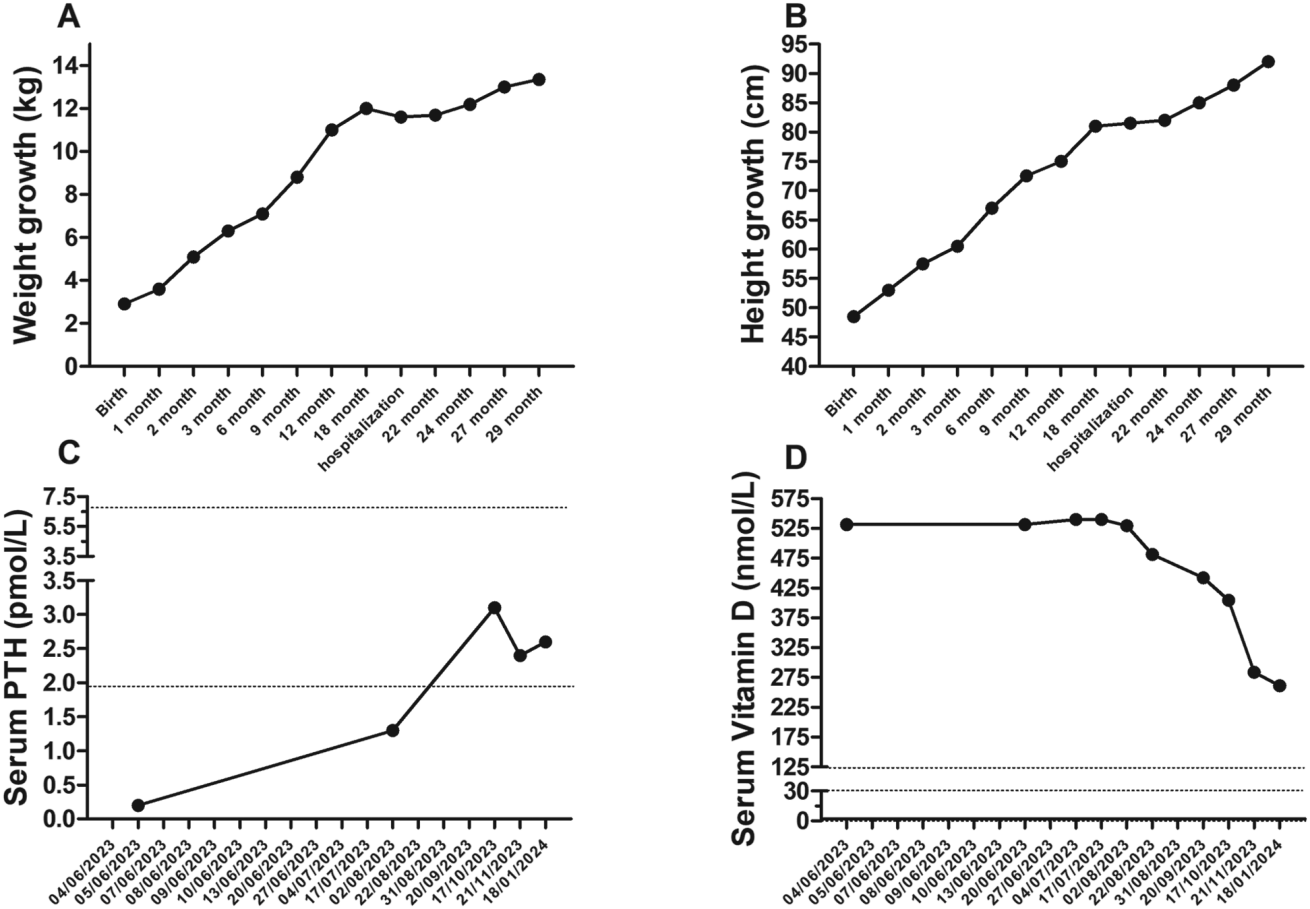
Serum PTH **(A)** and vitamin D levels **(B)**, weight **(C)** and height growth **(D)** over 29 months of follow-up.

**Figure 2:**
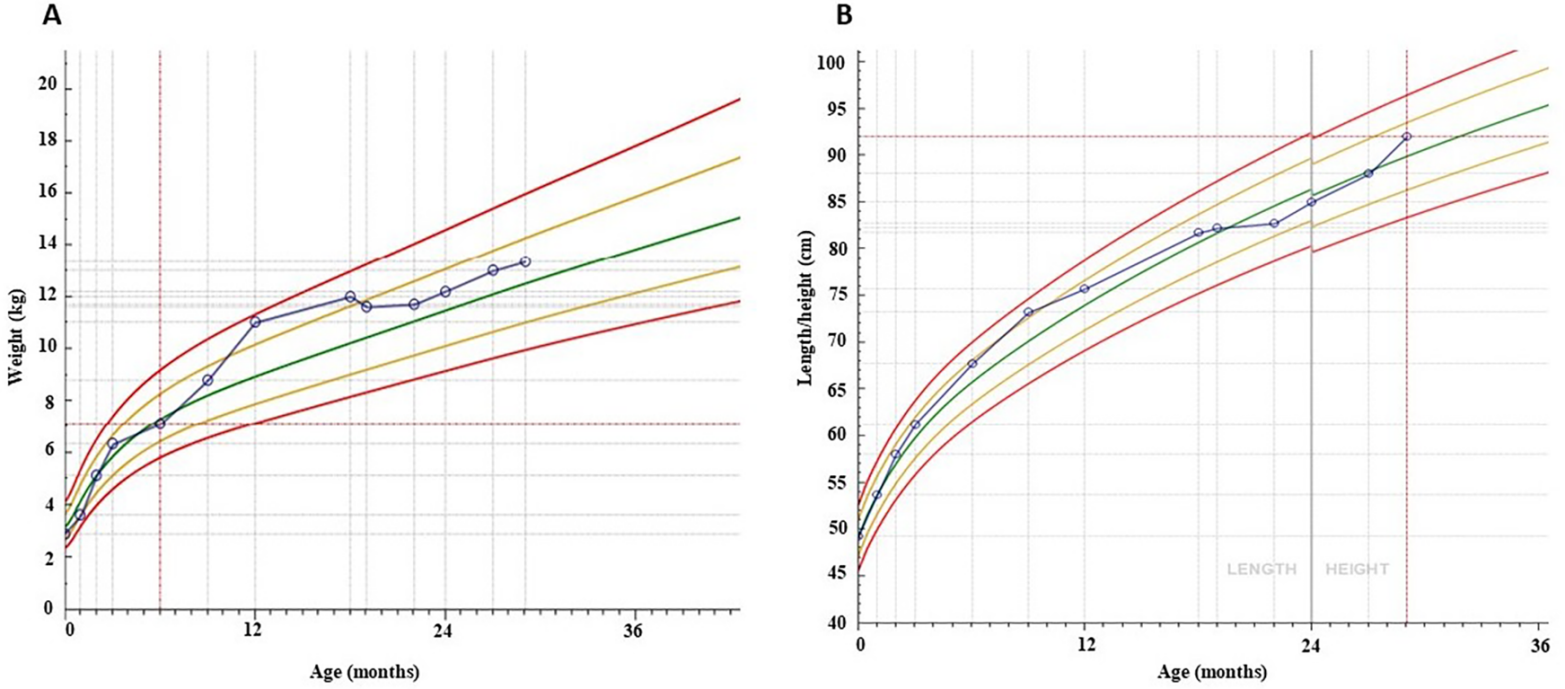
Weight-for-age **(A)** and length-for-age **(B)** chart over 29 months of follow-up.

We present the case of a 22-month-old girl who was brought to our pediatric emergency department by her father who expressed concerns about the vitamin D therapy: he believed it to be excessive. On 4 June 2023, the girl was admitted to the pediatric emergency department with a history of polydipsia, polyuria, and an increased fluid intake during the night (symptoms that have lasted for about 7 months). She also exhibited tiredness and a decreased growth rate (Figures 1C,D, 2).

Upon objective evaluation, the patient was found to be well-appearing with normal vital signs. A preliminary evaluation of the patient's condition ruled out the possibility of diabetes onset, without the need for further examinations. Following further anamnesis with the parents, it became evident that the infant had been administered an excessive dose of vitamin D supplementation. The pediatrician prescribed a vitamin D supplement of five drops per day, equating to approximately 400 IU daily. The supplement that parents had purchased online, however, had a higher concentration: 2,000 IU per drop, resulting in an unintended 25-fold increase in dose from the desirable level of 400 IU per day, administered for a period of seven months. (from November 2022 to June 2023). Patient's family history revealed no relevant disease.

### Diagnostic assessment

Laboratory tests conducted at the emergency department revealed that total calcium was within the normal range (total calcium 2.62 mmol/L; normal range: 2.25–2.75), ionized calcium and serum phosphate was slightly higher (respectively 1.34 mmol/L; normal range: 1.17–1.30 and 1.66 mmol/L; normal range: 1.12–1.45), parathyroid hormone levels were suppressed (0.2 pmol/L; normal range: 1.6–6.9), and that 25-hydroxyvitamin D concentrations were too high to be detected by available methods (>531.7 nmol/L; normal range: 75–250; Figures 1A–D, 3A,B). The renal function was within the normal range, as indicated by the values for urea, creatinine, and cystatin C. The urine examination detected pH 7, and the specific gravity 1,005. An electrocardiogram (ECG) was performed, which demonstrated no significant alterations. An ultrasound examination of the kidneys revealed the presence of typical hyperechogenic foci in the medullary pyramids, indicative of nephrocalcinosis (Figure 4A). In particular, the degree of nephrocalcinosis was described as at an initial phase in our patient.

**Figure 3:**
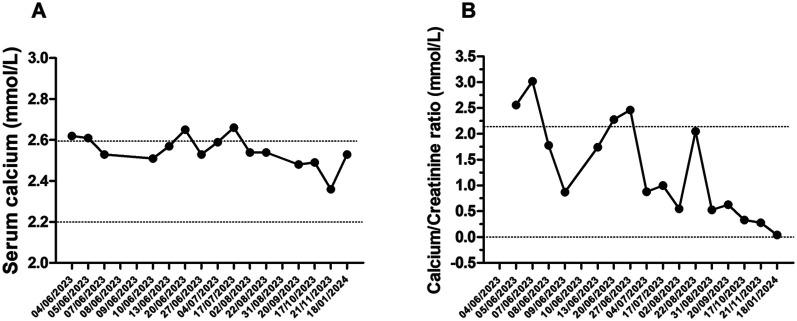
Serum calcium level **(A)** and calcium/creatinine ratio **(B)** over 29 months of follow-up.

**Figure 4:**
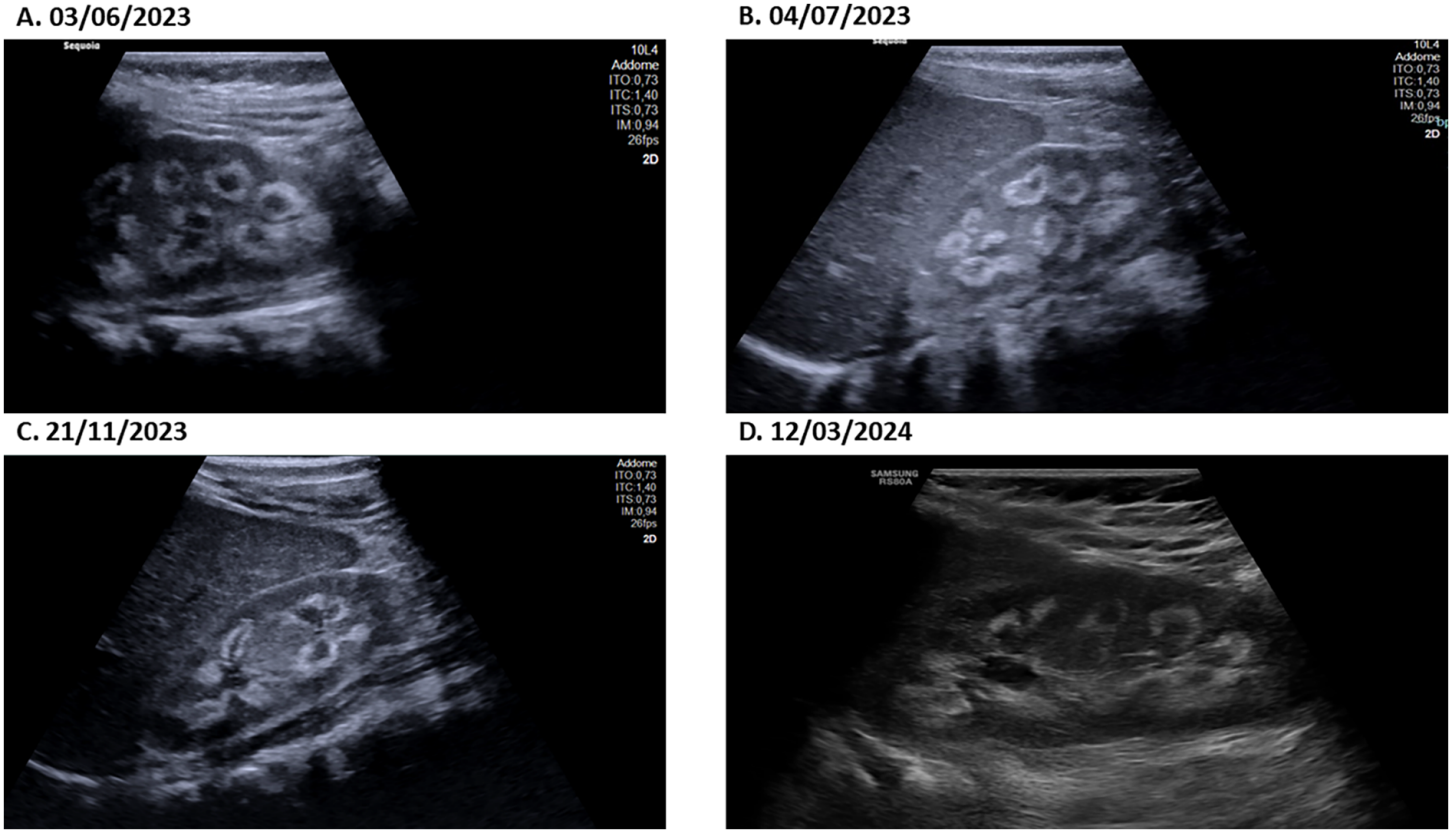
The ultrasound image of the patient's kidney at different time-points **(A–D)** definitively shows the presence of echogenic medullary pyramids, which are an unambiguous indicator of nephrocalcinosis.

### Therapeutic intervention and follow up

The patient was admitted to the hospital for the purpose of monitoring vital signs and evolution of biochemical urinary and serum electrolyte value. Intravenous hydration was initiated to stimulate urinary output and a thiazide diuretic was prescribed at a dosage of 1 mg/kg/daily in order to reduce hypercalciuria, both urinary calcium excretion and serum calcium levels was closely monitored until the values were stable. To prevent further crystallization, oral potassium citrate was started. A low-calcium diet was prescribed, with the aim of maintaining an adequate intake to ensure normal growth [approximately 450 mg/day (14)]. The administration of vitamin D therapy was obviously discontinued.

The patient was discharged on a course of hydrochlorothiazide and potassium citrate, with a follow-up appointment scheduled. The subject underwent clinical and haematochemical evaluations, including urinary and blood analyses, on a biweekly basis for three months, followed by monthly assessments for further eight months (Figures 1, 3).

One month following hospitalisation, the patient exhibited indications of recovery in terms of height and weight. Approximately one year after the diagnosis of vitamin D intoxication, the patient had almost fully recovered to her usual pre-intoxication growth curves. One year after the initial diagnosis, the patient exhibited normal growth and vitamin D levels. Serial ultrasound examinations revealed a persistently unchanged picture of nephrocalcinosis during the initial phase (Figures 4B–D). The patient was treated with thiazide and potassium citrate for a total of 11 months, after which the therapy was discontinued when the vitamin D levels returned to baseline.

## Discussion

Calcium and vitamin D status must be optimal in the first few years of life, as insufficient intake can cause nutritional rickets, hypocalcaemia and affect normal growth and development.

To date, there are several guidelines on calcium and vitamin D supplementation in healthy children throughout the world. Nevertheless, recommendations on the necessity, dosage and source of routine calcium and vitamin D supplementation in healthy children differ, complicating clinical decisions.

The 2010 IOM guidelines clearly state that 600 IU/day is the recommended intake for children, adolescents, and adults, while 400 IU/day is advised for infants (15, 16). The Endocrine Society asserts that infants up to one year old should receive 400–1,000 IU/day, while children older than one year should get 600–1,000 IU/day to treat and prevent vitamin D deficiency (17). These values are consistent with several other guidelines and should be increased if a laboratory-confirmed vitamin D deficiency is being treated (18).

In healthy individuals, hypervitaminosis D is typically “exogenous”, resulting from the unmanaged use of high doses of vitamin D or its metabolites and, as described in this report, such cases are usually caused by errors in the formulation, administration, and prescription of the medication (1). It is characterized by normal or reduced levels of Calcitriol, very low or undetectable levels of PTH and hypercalcemia (rarely severe, more often mild), hypercalciuria, and hyperphosphatemia. It is also important to consider that the impact of a specific dose on blood 25(OH)D levels differs significantly from one individual to another, due to various factors, including age, body weight, absorption, diet, CYP2R1 activity, and other metabolism genetics (18).

The dosage of vitamin D taken by our patient was lower than the toxicity range reported by other similar case reports, ranging from 50,000 IU/day–2,604,000 IU/day (1). However, the patient's severe toxicity only resolved approximately a year after treatment started.

This case report emphasizes the potential risks of taking such supplements without proper knowledge and guidance and as previously reported by Kaptain et al. (10), some over-the-counter supplements may contain higher levels of vitamin D than stated on the label, leading to serious complications. For these reasons, it is crucial for consumers to consult a healthcare professional before taking supplements. They must also strictly adhere to the manufacturer's recommended dose and usage instructions. Meanwhile, doctors must be more aware of the potential risks of vitamin D toxicity. Indeed, vitamin D intoxication can cause hypercalcaemia, which must be treated promptly to avoid life-threatening complications such as renal failure, pancreatitis and cardiac arrhythmia. This is a serious issue that must be carefully considered, despite it being rare and usually occurring with very large amounts of vitamin D supplementation.

Finally, adequate fluid intake and dilution of urine are the keys to prevent nephrocalcinosis progression. Thiazide diuretics and potassium citrate, a natural inhibitor of calcium oxalate stones, respectively reduce the risk of recurrence of calcium oxalate stones by about 50% and calcium excreted in urine (12). It's important to stress that thiazide diuretics reduce the worsening of nephrocalcinosis through the reduction of hypercalciuria, but they can cause an increase in tubular calcium reabsorption, therefore it is necessary to carefully monitor the correct intake of the drug and periodically check the calciuria and calcium values.

## Conclusions

Despite the wide therapeutic index of vitamin D, this report highlights the crucial need for healthcare professionals to be aware of the potential risks associated with prescribing and administering errors, particularly in pediatric populations. This is particularly important given the variety in terms of quality of food supplements offered on the market. It may be beneficial for pediatricians to consider providing parents with a list of safe and appropriate over-the-counter vitamin D supplements, particularly for infants and toddlers. Concurrently, it would be prudent for policymakers to mandate the inclusion of paediatric warning labels on all products available for purchase.

Finally, in the literature there are no detailed paper to our knowledge that describe the long-term evolution of nephrocalcinosis in children with vitamin D intoxication. Given the rarity of cases of intoxication in pediatric age it would be very important to establish a database that can be consulted in order to apply appropriate therapies and to give parents information about the long-term outcomes of intoxication.

## Data Availability

The raw data supporting the conclusions of this article will be made available by the authors, without undue reservation.
